# Flow Cytometric Enumeration of Parasitemia in Cultures of *Plasmodium falciparum* Stained with SYBR Green I and CD235A

**DOI:** 10.1155/2014/536723

**Published:** 2014-12-08

**Authors:** Jin Woo Jang, Ju Yeon Kim, Jung Yoon, Soo Young Yoon, Chi Hyun Cho, Eun Taek Han, Seong Soo A. An, Chae Seung Lim

**Affiliations:** ^1^Department of Laboratory Medicine, Brain Korea 21 Graduate School of Medicine, College of Medicine, Korea University, Seoul 136-703, Republic of Korea; ^2^Department of Parasitology, Kangwon National University College of Medicine, Chuncheon, Kangwon-do 200-701, Republic of Korea; ^3^College of Bionano Technology, Gachon Bionano Research Institute, Kyungwon University, Seongnam-si, Kyeonggi-do 461-701, Republic of Korea; ^4^Department of Laboratory Medicine, College of Medicine, Korea University, Guro Hospital, Guro 2 Dong, Guro Gu, Seoul 152-703, Republic of Korea

## Abstract

A flow cytometric (FACS) detection method for *Plasmodium falciparum* cultures (*P. falciparum*) was developed using SYBR Green I and CD235A and compared against the Giemsa stained microscopic examination. The cultured *P. falciparum* were spiked into red blood cells (RBCs) to yield parasitemia, ranging from 0.01% to 22.0%. FACS analysis demonstrated a clear separation between *P. falciparum* infected and uninfected RBCs. The measured percentage of parasitemia by FACS revealed higher precision (CV of 2.2–37.2%) with the sensitivity of 0.01% parasitemia than Giemsa stained microscopic examination (CV of 7.2–66.0%). High correlation of measured parasitaemia (*r* = 0.98, *P* < 0.05) was observed between FACS and Giemsa stained microscopic analyses. The higher levels of parasitaemia detection were observed in all ranges by FACS in comparison to Giemsa stained microscopic analysis. The currently reported FACS method using SYBR Green I and CD235A is potentially useful for measuring parasitemia in treating patients.

## 1. Introduction

Malaria is the most important infectious disease in tropical and subtropical countries with high morbidity, mortality, and extensive economic loss [1]. Despite many new efforts to curve the transmission of malaria over the past decades, the disease continues to be one of major health problems [2].

Until now, the diagnosis of malaria usually depended upon an expert reading of Giemsa stained thin and thick peripheral blood smears, despite many technical disadvantages [3]. The PCR molecular detection and immunochromatographic methods were proven to be excellent diagnostic approaches with high efficacy. Special expensive PCR instrument with trained personnel became the limitation for the use of PCR [4], while the rapid immunochromatography showed lower sensitivity than both PCR and traditional Giemsa stained methods [5]. Hence, no single technique with fast diagnosis and monitoring drug treatment of patients could replace the traditional Giemsa stained microscopic method.

In addition, efficient control and screening of malaria over relatively large numbers of suspected persons could require methods with high sensitive and quantitative techniques, especially with rapid diagnosing time. Enumeration of parasitemia by semiautomations or full automations could become important tools to evaluate and follow the progression of malaria [6].

Flow cytometry (FACS) was established as a reliable, precise, and fast method for the measurement of parasite load in human blood samples or in malaria cultures at a routine laboratory setting [6–10]. It could also count the number of parasites and evaluate the malaria-infected red cells. In previous reports, different dyes such as Hoechst 33258 [11], acridine orange [12], thiazole orange [13], or hydroethidine [14] were used to determine parasitemia in cultures of* Plasmodium falciparum* by FACS. Recently, asymmetric cyanine nucleic acid dyes, SYTO and YOYO series, became popular [15, 16] with the coefficiency of variation (CV) at 1.20% and 11.56% for 37.54% and 0.2% parasitemia, respectively.

This study demonstrated a practical dual stain protocol with SYBR Green I (Molecular Probes Inc., Oregon, USA) and CD235A (BD Biosciences, USA) in FACS enumeration of parasitemia, which could be used in routine clinical laboratories with high precision and efficiency. The results were analyzed in comparison against the Giemsa stained microscopic examination. Consequently, a quick and reliable evaluation method of parasitemia was developed with cultured* P. falciparum*.

## 2. Materials and Methods

### 2.1. *P. falciparum* Culture

Laboratory line 3D7* P. falciparum* malaria parasites were grown with human erythrocytes (group O, Rh-positive, 3% hematocrit) in RPMI-HEPES medium supplemented with 40 mg/L gentamicin (Invitrogen Co., USA), 1.36 g/L hypoxanthine (Sigma Aldrich, USA), 25 mM HEPES (Sigma Aldrich, USA), 7.5% sodium bicarbonate (Invitrogen Co., USA), 20% glucose (Sigma Aldrich, USA), 1 M NaOH (Sigma Aldrich, USA), and 20% Albumax (Invitrogen Co., USA), as previously described [17]. All cultures were maintained at 37°C in an atmosphere of 5% CO_2_, 1% O_2_, and 94% N_2_, with daily medium changes [17]. Synchronization of culture was achieved through sorbitol lysis at mature stage using 5% sorbitol (Sigma Aldrich, USA) and fine-tuned by another lysis after 8 hours [18].

### 2.2. Sensitivity of Detection

To determine the sensitivity of the detection, cultured malaria samples were spiked into 3% suspension of uninfected erythrocytes (RBCs) and serially diluted by twofold. The malaria-infected RBCs with 44% parasitemia were diluted with blood from an uninfected donor to obtain parasitemias, ranging from 0.001 to 22.0%. Each serially diluted sample was then analyzed in triplicate with FACS and Giemsa stained microscopic examinations. The detection limit was determined by counting the number of parasites in a corresponding dilution.

### 2.3. Microscopic Determination of Parasitemia by Giemsa Stained Smear

Thick and thin blood films were stained with 5% Giemsa. Malaria parasites in various developmental stages were counted in the presence of 200 WBCs in thick blood films, or the percentage of parasitemia was calculated against 1,000 RBCs in thin blood films. Parasite density (parasites per *μ*L of blood) was calculated by comparing against the actual WBC or RBC counts per *μ*L.

### 2.4. Fluorescent Staining and Parasitemia Determination on Wet Smear

The fluorescent dye, SYBR Green I (supplied as a 10,000x concentrate in dimethylsulfoxide, DMSO), was diluted with 10% ethanol to 1 : 1,000 ratio as a working concentration.* P. falciparum* cultured samples (50 *μ*L, 3% haematocrit) were mixed with the same volume of dye solution and incubated in the dark for 20 min at room temperature. After washing with 500 *μ*L PBS, a drop of stained* P. falciparum* cultures was placed directly onto the slide with a cover slide. The wet blood films were examined using Olympus BX61 fluorescence microscope under 1000x magnification. Filter sets included DAPI, CFP, GFP, YFP, and Texas Red.

### 2.5. Red Blood Cell Preparation for Flow Cytometry

*P. falciparum* infected RBCs were mixed with 1% paraformaldehyde solution in PBS buffer at various concentrations and stored at 4°C for 30 min. The paraformaldehyde fixed RBCs were washed with PBS and centrifuged three times at 450 g for 5 min. After aspirating supernatant, (1) 50 *μ*L of washed sample was incubated with 50 *μ*L of 1 : 1,000 diluted SYBR Green I (Invitrogen, USA) and 20 *μ*L of CD235A-PE (BD Pharmingen, Becton Dickinson Biosciences, Franklin Lakes, NJ) for 15 min at room temperature. (2) For the antibody stain, 50 *μ*L of washed sample was incubated with 20 *μ*L of Anti-H (IQ Products, Netherlands) and 50 *μ*L of 1 : 1,000 diluted Propidium Iodide solution (5 mg/mL) for 15 min at room temperature. Before FACS analysis, stained samples were washed twice with PBS and resuspended in 1 mL of PBS.

### 2.6. FACS Analysis

FACS data acquisition and analysis were performed on a FACS Calibur equipped with standard filters and CELLQuest software (BD Biosciences, USA). Forward light scatter (FSC) and side scatter (90°) (SSC) detected the samples simultaneously with green fluorescence (FL1) and red fluorescence (FL2), respectively. At least 5,000 particles were assessed and plotted in two-dimensional scattergrams of two of these four parameters, FSC, SSC, FL1, and FL2. The parasite areas, RBCs area and parasite infected RBCs, were detected by analyzing scattergrams from the computer software.

### 2.7. Statistical Analysis

The sensitivity, specificity, accuracy, and linearity of the FACS were calculated and compared against Giemsa stained microscopic examination as the gold standard test for detecting malaria parasites. Pearson's correlation test was used to compare two tests.

## 3. Results

### 3.1. Staining of* P. falciparum* Cultures with SYBR Green I

**Figure 1:**
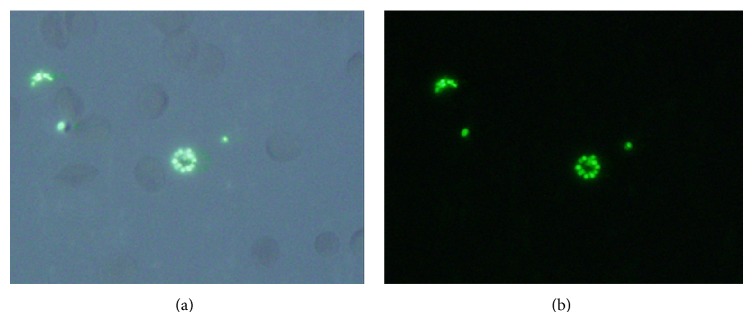
*P. falciparum* infected RBCs were stained with SYBR Green I and were then examined using a microscope with (a) DAPI and (b) fluorescence filter. Photographs indicated fluorescent images of parasites at schizont (center) and merozoite stages (outer).

Staining malaria parasites in RBC with SYBR Green I fluorescence dye allowed clear visualization with one of two standard filter sets: Ex. 340–380, BA 435–485 (Fluorescein); Ex. 450–490/Em 520 (DAPI). The optimal concentration of SYBR Green I for the above conditions was found to be at 1 : 1,000 dilution (Figure 1).

### 3.2. Comparison of Giemsa Stained Microscopy and FACS Assessments of* P. falciparum* Parasitemia

**Figure 2:**
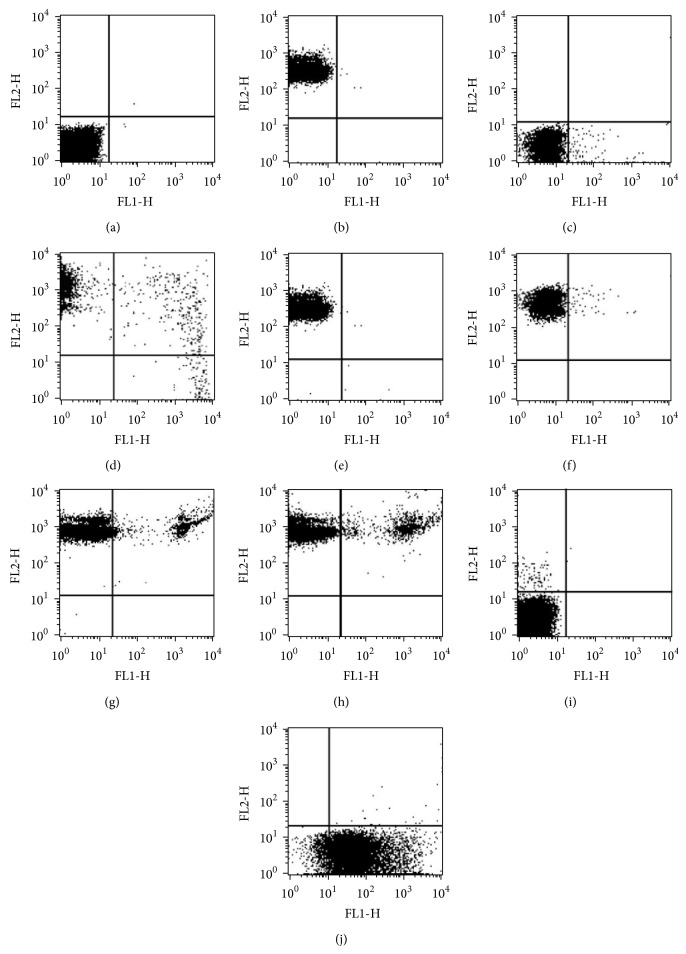
Representative two-channel (FL-1/FL-2) dot-plots, examining various levels of parasitemia. Cultures of* P. falciparum* were stained with SYBR Green I and CD235A (a–h), and Propidium Iodide and Anti-H (i-j). Over 5,000 events were acquired for each dotplot. (a) Normal unstained RBCs were plotted on the lower left corner. (b) Anti-CD235A PE stained RBCs were plotted between 10^1^ and 10^3^ on the red axis with small diagonal stretch from the upper left corner (FL2). (c)* P. falciparum* infected RBC populations were plotted parallel to the noninfected RBC between 10^1^ and 10^4^ on the green axis (FL1). (d)* P. falciparum* infected RBC populations controls stained with SYBR Green I and CD235A. Suspected external merozoites from RBCs were localized on the right lower quadrant. Synchronized cultures of* P. falciparum* with parasitemia were used. (e) 0.01%, (f) 0.5%, (g) 10.0%, and (h) 22.0%.

**Figure 3:**
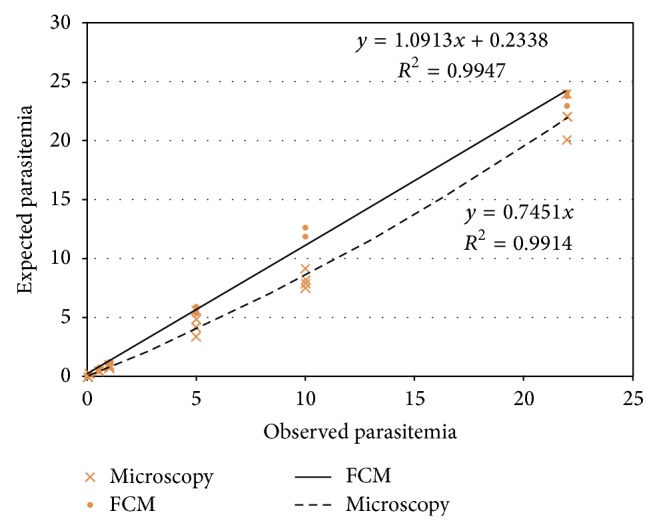
Correlation between FACS and Giemsa stained microscopic determinations of parasitaemia with artificially diluted samples. The lines represented the linear regression whose statistical parameters were shown in the inset. Each symbol represented FACS measurement of parasitemia (in 10,000 RBCs) and its corresponding microscopic manual count. Plotted parasitemias ranged from 0.01% to 22.0% (expected values).

FACS analysis with SYBR green I demonstrated a clear separation of RBCs with* P. falciparum* infection from uninfected RBCs (Figures 2(a), 2(b), and 2(c)). FACS counts gave higher values (122%) in comparison with the Giemsa stained microscopic examination. In addition, the coefficient variance (CV) was lower in FACS (12.6%) than Giemsa stained microscopy (23.4%). The percentage of parasitemia between both methods showed strong correlation as indicated in Figure 3 from Pearson's correlation tests (*r* = 0.98, *P* < 0.05). The sensitivity of the FACS analysis by SYBR Green I and CD235A was 0.01%, measured as the percentage of detectable parasitemia above the background level. An accurate and linear correlation between FACS and Giemsa stained microscopic enumeration of parasitemia was found, ranging from 1% to 20% (*r* ^2^ = 0.99). SYBR Green I and CD235A dual staining could not distinguish the quantitative differences in the nuclear content of* P. falciparum* (Table 1). In contrast to SYBR Green I and CD235A stain, Propidium Iodide stain with Anti-H did not show clear separation between infected and uninfected RBCs with* P. falciparum* (Figures 2(i) and 2(j)).

## 4. Discussion

Microscopic examination of Giemsa stained smear is the time-honored method for the laboratory confirmation of malaria, including species identifications. Enumeration of malaria parasitemia is also important to detect drugs resistance during treatments. Identifications of various developmental stages of parasites depend heavily on the morphologic changes, requiring skilled observers and extensive observation time.

FACS was proven to be a very powerful tool in malaria research. It could identify various developmental stages of* P. falciparum*, based solely on nucleic acid content, without recourse to morphologic changes [9, 19]. For the enumeration of parasitemia in blood samples, FACS offered higher precision and efficiency than the manual Giemsa stained microscopic counting [15]. Since nucleic acids were present in malaria-infected RBCs but not in normal ones, FACS measurement of parasitemia in whole blood was based on the utilization and the detection of the fluorescent nucleic acid intercalating dyes, such as SYBR Green I, PicoGreen, and YOYO-1 [12–16].

In our study, the mean parasitemia determined with FACS gave slightly higher values in comparison to Giemsa stained microscopic data, which was similar to previous reports with YOTO-1 [8, 10, 15, 16], acridine orange [20], and Propidium Iodide [19]. The results indicated that FACS detected infected RBCs with higher sensitivity than Giemsa stained microscopic analysis, even in weakly expressed stages of parasites.

Recently, SYBR Green I, PicoGreen, and YOYO-1 were also used to measure the malaria growth inhibition* in vitro* [21–23]. Among the above dyes, the data from SYBR Green I in malaria drug surveillance gave better results than others in monitoring the presence of residual parasites [22]. Hence, we employed the particular dual staining method (SYBR Green I and CD235A) of enumerating* P. falciparum* infected RBCs because CD235A could give reliable RBC gating, comparable to those obtained by conventional microscopy methods. After staining with both SYBR Green I and CD235A, few signals were found from the right lower quadrant (Figure 2(e)), which suspected the presence of merozoites in Figure 1. Merozoites are not localized within RBCs, while trophozoites, gametocyte, and schizonts are known to be attached to the RBC membranes. Therefore, signals detected in right upper quadrant would reflect the presence of DNA contents in various malaria stages (Figures 1, 2(g), and 2(h)). SYBR Green I fluorescence intensities correlated with increases in parasitic DNA contents [23]. Schizonts also seemed to cover RBC membrane, which supported the previous reports [24].

Our FACS experiments with SYBR Green I and CD235A revealed clear background signal compared to YOYO-1 (0.05–4%) [8, 10, 15, 24]. Excellent background levels in the present study lowered the detection limit to 0.1% parasitemia from the dilution test. Therefore, FACS with SYBR Green I and CD235A established the acceptable and practical limits of sensitivity and could be used routinely for evaluating the antimalarial drug resistance in clinical laboratories. Problems associated with the use of SYBR Green I and CD235A could be the loss of merozoites during washing process, resulting in the reduction of real parasitemia and the close correlation with the parasite growth stages. Differentiating between the various human malaria species was not possible until now. Due to fixation process during stain, this assay did not allow the differentiation between living and dead parasites. However, it has many advantages such as displayed cellular information, reliability, and ability of automation. Therefore, when measurement conditions are optimized, this flow cytometry should be very helpful in all areas related to malaria diagnosis, treatment, and researches.

In summary, a FACS assay with SYBR Green I and CD235A was developed for measuring parasitemia of malaria-infected RBCs, which could be used for monitoring the drug treatments. This method also offered a specific, sensitive, and inexpensive approach to differentiate malaria-infected RBCs, avoiding potential bias associated with Giemsa stained microscopic manual counting.

## Figures and Tables

**Table 1:** Comparison of FACS with SYBR Green I and Giemsa stained microscopic analyses for the determination of malaria parasitemia.

Expected parasitemia (%)	Observed value						FACS/microscopy (%)
	Light microscopy			FACS			
	Mean	SD	CV (%)	Mean	SD	CV (%)	
22	22.05	1.59	7.20%	23.68	0.53	2.20%	107%
10	8.23	0.69	8.40%	12.45	0.37	3.00%	151%
5	4.51	0.92	20.50%	5.63	0.25	4.40%	125%
1	0.89	0.17	18.70%	1.18	0.13	10.70%	132%
0.5	0.52	0.05	9.00%	0.68	0.1	14.60%	132%
0.1	0.17	0.06	33.70%	0.15	0.02	16.30%	91%
0.01	0.01	0.0096	66.00%	0.02	0.0062	37.20%	116%
